# The Coexistence of Trachyonychia and Mucocutaneous Lichen Planus: A Case Report

**DOI:** 10.7759/cureus.48415

**Published:** 2023-11-06

**Authors:** Kaveri Rusia, Adarshlata Singh, Bhushan Madke, Sugat Jawade

**Affiliations:** 1 Dermatology, Jawaharlal Nehru Medical College, Datta Meghe Institute of Higher Education and Research, Wardha, IND

**Keywords:** classic lichen planus, hypertrophic lichen planus, nail diseases, trachyonychia, oral lichen planus

## Abstract

Trachyonychia is an uncommon finding characterized by the nails having a uniform and simultaneous presence of fragility, excessive longitudinal growth, ridging, and loss of luster. Usually, twenty-nail dystrophy (TND)/trachyonychia is an idiopathic condition, but sometimes dermatoses such as alopecia areata, lichen planus, and psoriasis are found to be associated with it. We report a case of trachyonychia/TND in a young male with the concomitant presence of cutaneous lichen planus of hypertrophic type, reticular oral lichen planus, and nail lichen planus, which was diagnosed with the aid of dermoscopy and histopathology. Many cutaneous disorders, systemic illnesses, and infections can cause nail dystrophy; therefore, a proper diagnosis is crucial to treat the underlying cause. Early intervention improves patients' prognosis and alleviates their psychological strain and cosmetic concerns.

## Introduction

Lichen planus refers to a benign, long-term mucocutaneous condition affecting the skin, mucosae, and nails, which clinically presents as violaceous, pruritic, flat-topped, polygonal, papules, and plaques. It has several variants in terms of morphology and location, including oral, nail, linear, annular, atrophic, hypertrophic, inverse, eruptive, bullous, ulcerative, lichen planus pigmentosus, lichen planopilaris, vulvovaginal, actinic, lichen planus-lupus erythematosus overlap syndrome, and lichen planus pemphigoides [1].

Cutaneous lichen planus is an uncommon dermatosis that affects people of all ethnicities and genders equally. While its prevalence ranges from 0.22 to 1%, the more prevalent oral illness affects 2-5% of the overall population, with females experiencing it twice as frequently as males. Although the skin and oral mucosa account for the majority of cases of lichen planus, other mucous membranes and skin appendages, like nails and hair, can also be involved [2].

Nail lichen planus has been observed to occur in 10-15% of cases [3]. Based on whether the nail lichen planus is located in the nail matrix or the nail bed, several abnormalities of the nail might be identified. One classic and permanent finding in nail lichen planus is dorsal pterygium. Trachyonychia is a disease with an insidious course that primarily affects children [4]. All 20 nails are often impacted in children, and idiopathic trachyonychia is more common. Trachyonychia has been associated with many conditions like lichen planus, alopecia areata, psoriasis, and amyloidosis [5]. Trachyonychia and nail lichen planus can coexist or each may present as an individual entity. The presentation of nail lichen planus caused by trachyonychia includes nail plate thinning, splitting of nails, or atrophy; pterygium formation and scarring are less common [6].

## Case presentation

**Figure 1:**
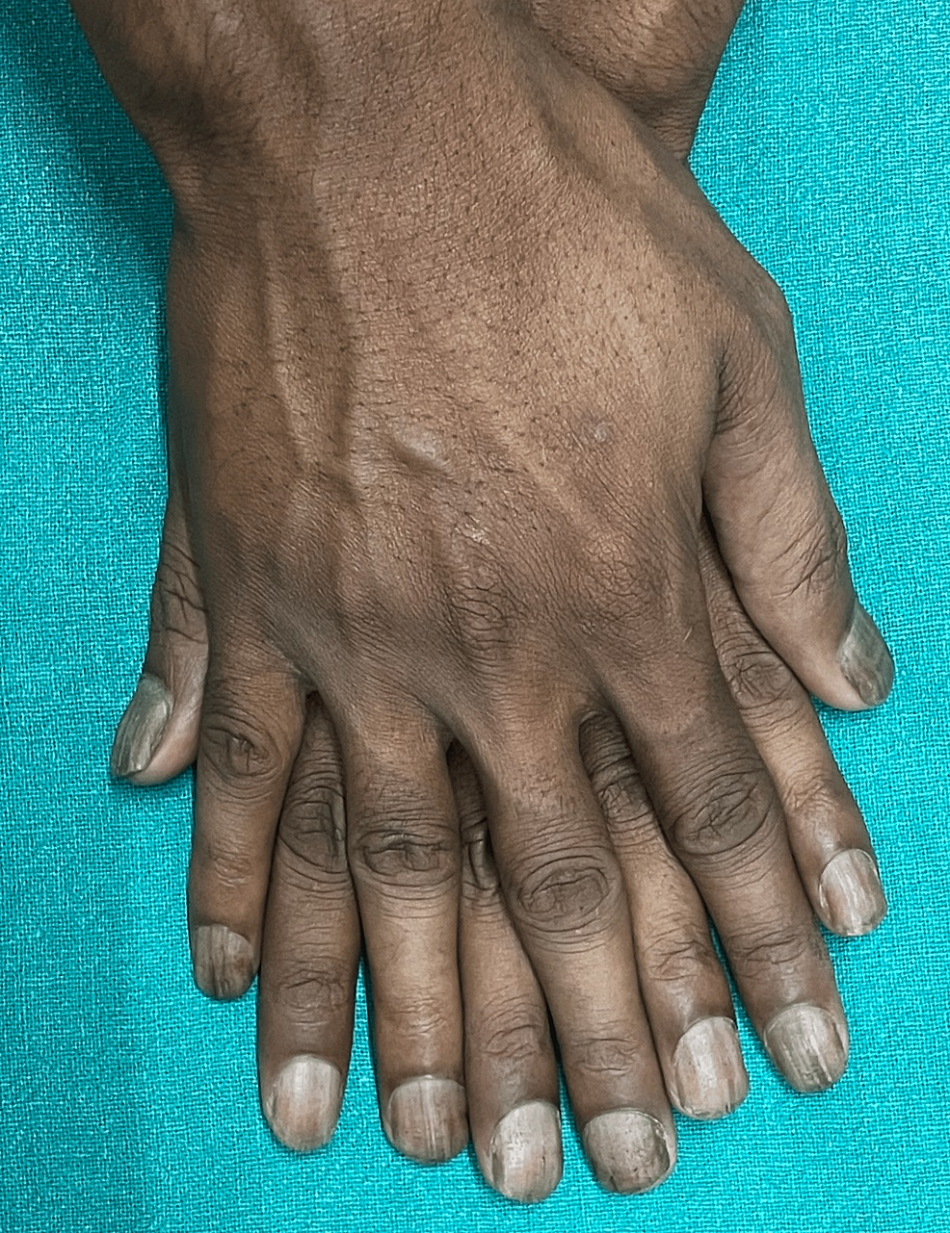
Longitudinal ridging of the nail plate in fingernails

**Figure 2:**
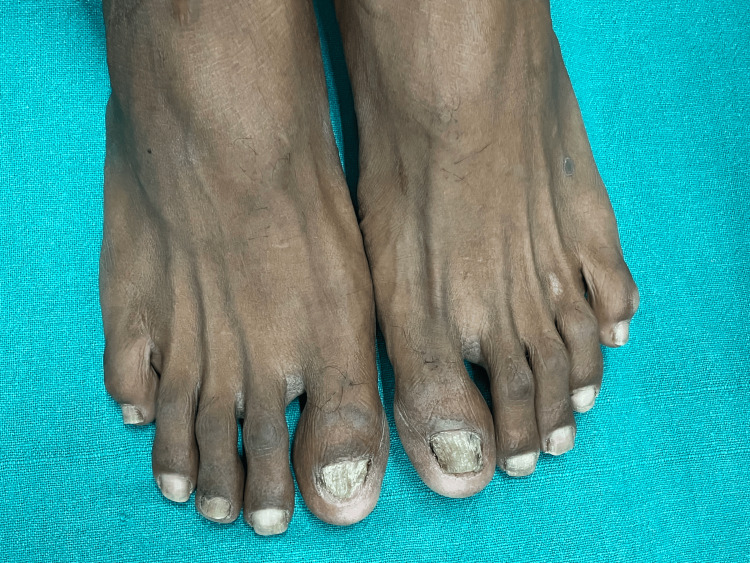
Longitudinal ridging of the nail plate in toenails

A 28-year-old male from central India presented to the outpatient department of a tertiary care hospital with complaints of roughness, easy-breaking, and flattening of all nails of fingers and toes for eight months. Initially, the patient had developed roughness of only two nails of the toes, which had later progressed over a period of eight months to involve other nails of fingers and toes. Clinical examination of nails of fingers and toes showed muddy grayish-white discoloration with alternating elevations and depressions (ridging), pitting, lack of luster, sandpaper-like roughness, and splitting (Figures 1, 2).

**Figure 3:**
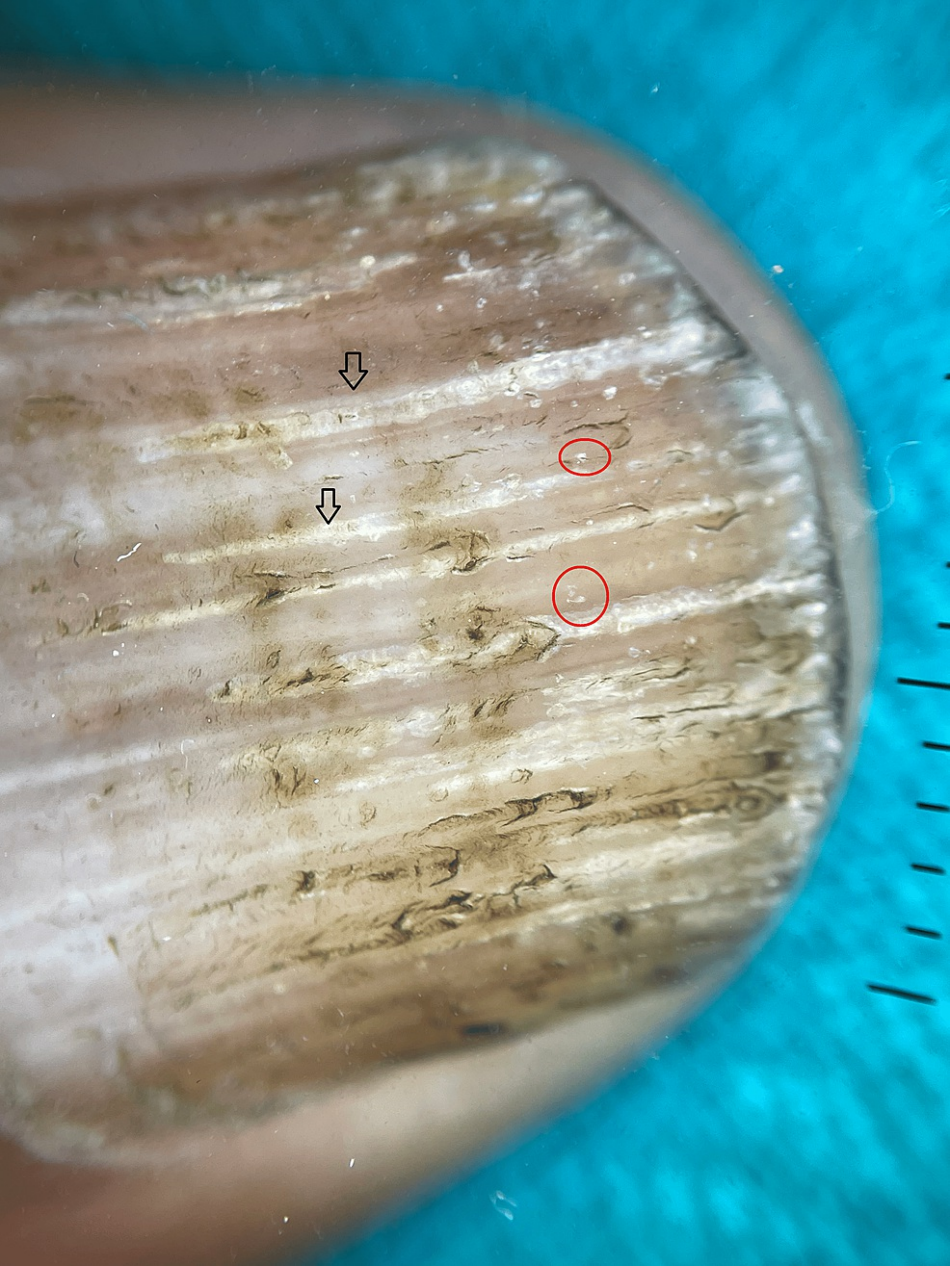
Dermoscopy of thumbnail shows longitudinal ridging (black arrows) suggestive of trachyonychia and nail pitting (red circle)

On polarized dermoscopy of the thumb, the presence of longitudinal ridging suggestive of trachyonychia and nail pitting was observed (Figure 3).

**Figure 4:**
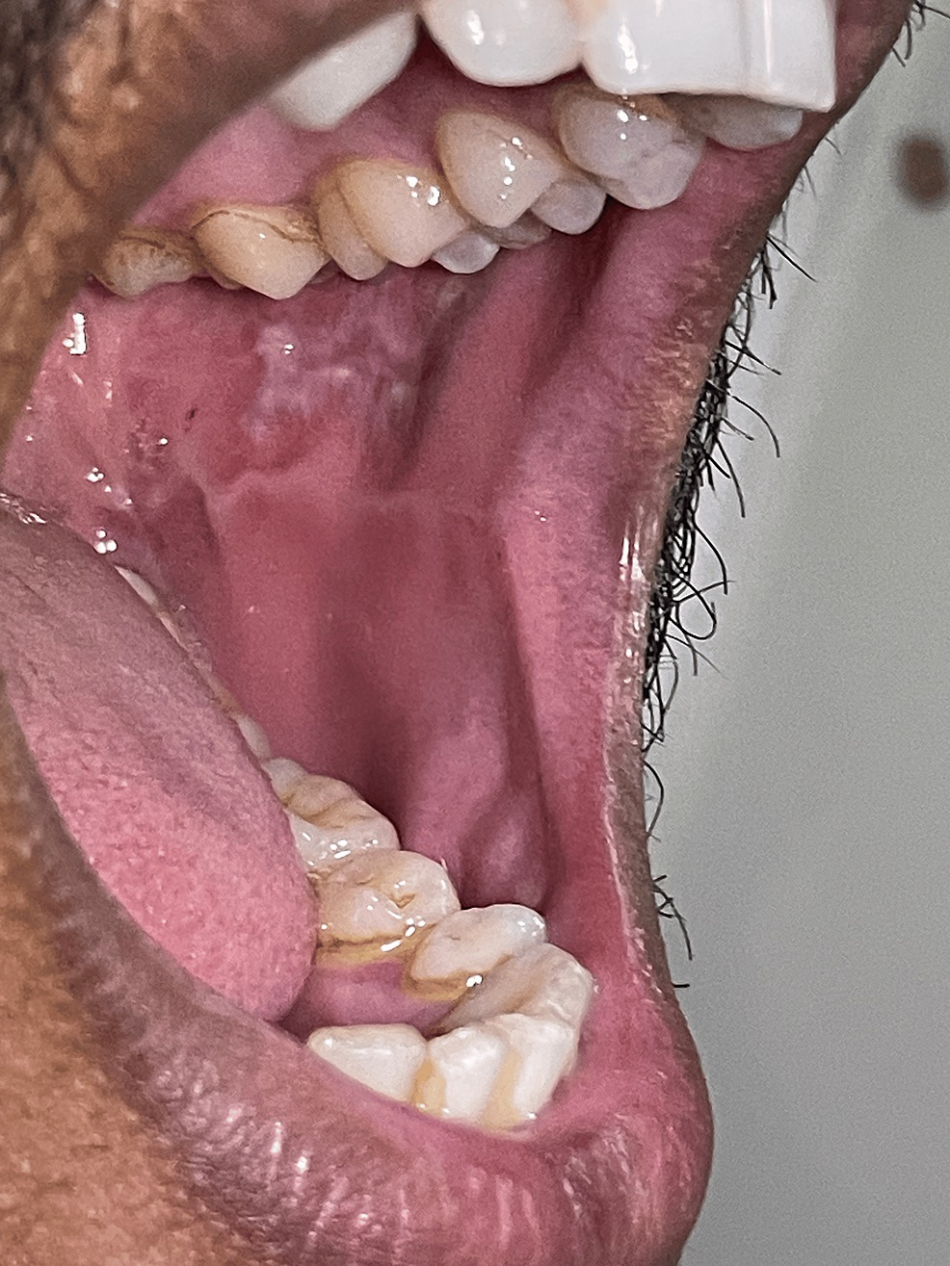
Characteristic Wickham's striae are present on both buccal mucosa, suggestive of the reticular type of oral lichen planus

The patient did not have any other complaints. On general physical examination, the oral cavity had multiple white streaks present on both buccal mucosa, suggestive of a reticular type of oral lichen planus (Figure 4).

**Figure 5:**
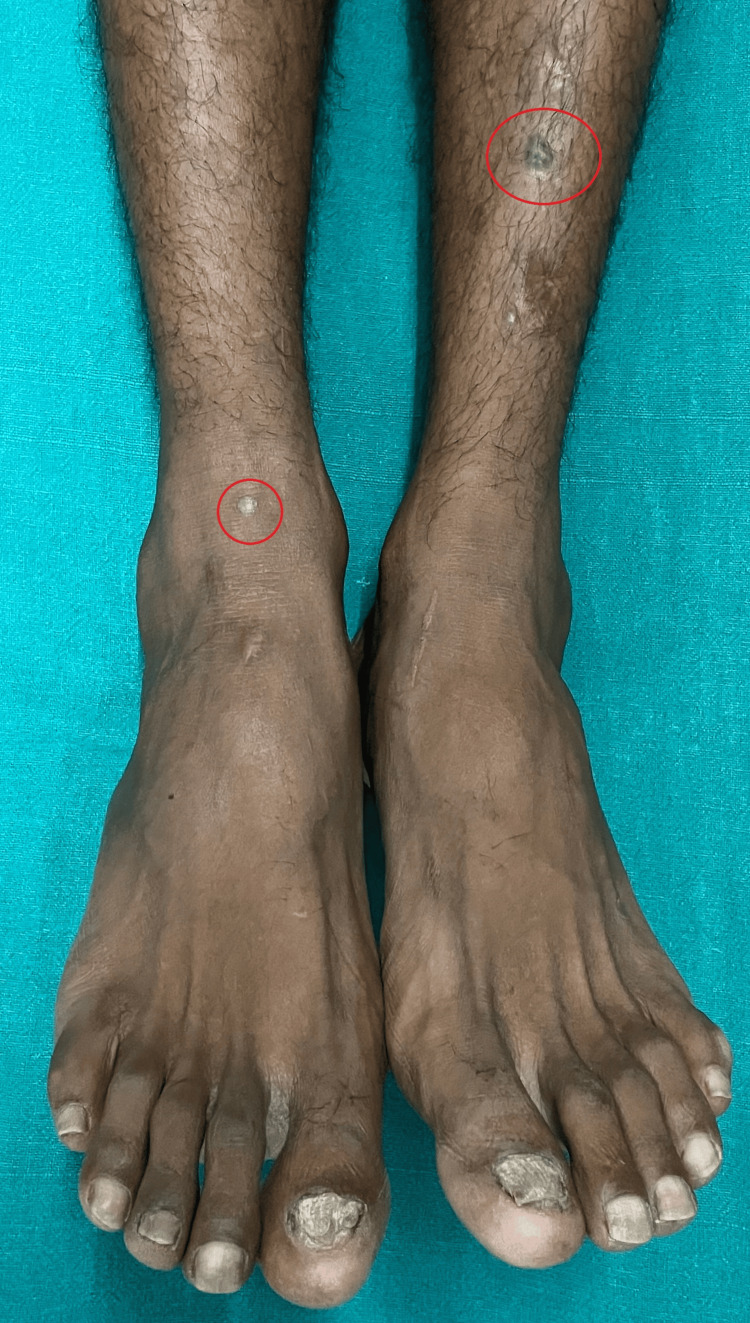
Hyperkeratotic plaques are seen on both the lower limbs (red circles) with ridging toenails

On cutaneous examination, the presence of violaceous flat-topped plaques on both the lower limbs was noted (Figure 5).

**Figure 6:**
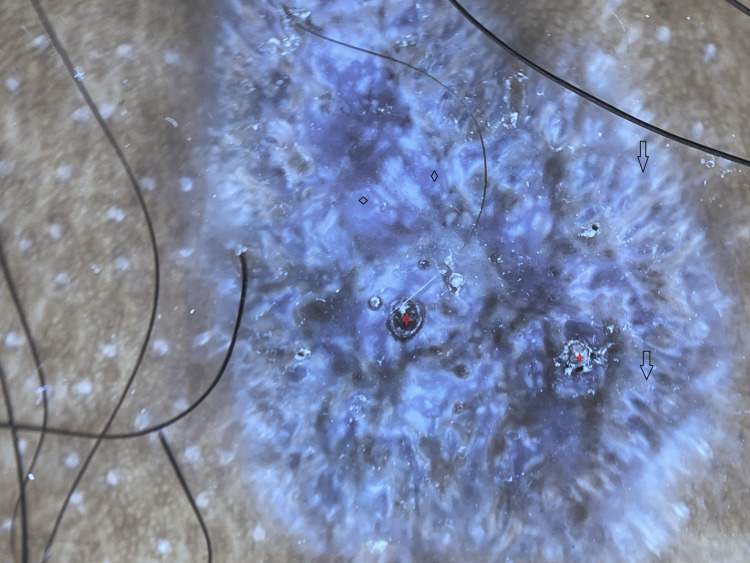
Dermoscopy of hypertrophic lichen planus shows peripheral striation (black arrows), gray-blue globules (black diamond), comedo-like openings (red four-point star) with violaceous background and diffuse brown pigmentation

A dermoscopy was done, which showed multiple comedo-like openings in a violaceous background with peripheral reticular striations suggestive of the hypertrophic type of lichen planus (Figure 6).

**Figure 7:**
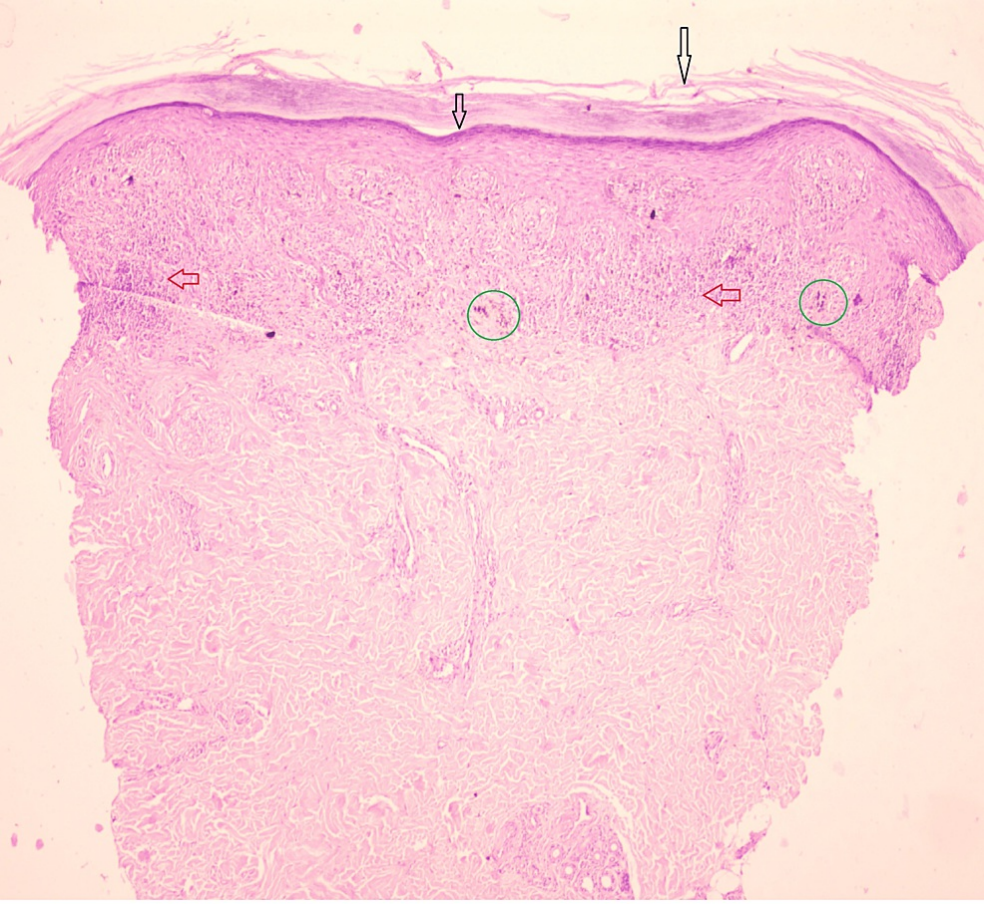
H&E section of skin tissue showing hyperkeratosis, hypergranulosis (black arrows), band-like lymphocytic infiltrate (red arrows), and melanin incontinence (green circle)

There was no involvement of other flexural areas, genital mucosa, or scalp. On the basis of the findings of clinical examination and dermoscopy, the differential diagnosis included lichen planus, twenty-nail dystrophy (TND), onychomycosis, and psoriasis. Routine laboratory investigations such as complete blood counts, renal function tests, and liver function tests were done and were within normal ranges. Tests for hepatitis B and hepatitis C were negative. To confirm the diagnosis, potassium hydroxide (KOH) from nails was done to rule out dermatophytes and yeasts, which was negative, and a 4-mm punch biopsy was done to rule out the differential diagnosis. A skin biopsy was taken from the left lower limb, which showed thickened stratum corneum, hypergranulosis, band-like infiltration of lymphocytes, and melanin incontinence (Figure 7).

On the basis of clinical, dermoscopic, and histopathological findings, we confirmed the diagnosis of hypertrophic lichen planus with trachyonychia/TND. We treated the patient with tofacitinib 5 mg tablets bd and clobetasol propionate 0.05% ointment twice daily.

## Discussion

Lichen planus is an inflammatory disorder with a chronic course affecting skin as well as mucosa. It is more common among adults than in children. It has several clinical variants with different morphological characteristics and clinical presentations [1]. In our case, there was a concomitant presence of oral reticular, cutaneous hypertrophic, and nail lichen planus along with TND/trachyonychia. There is a complex interplay of environmental factors, genetic susceptibility, and immune-mediated mechanisms in the development of lichen planus. The initial stage of lichen planus inflammation involves an antigen-directed response, which ultimately leads to effector T cell development and activation. The progression phase is carried out by the T helper and T cytotoxic cells, which ultimately leads to the generation of free radicals, damage to basal keratinocytes, and cell apoptosis [2]. Hypertrophic lichen planus is characterized by hyperkeratotic purple-to-gray plaques with follicular accentuation. These plaques typically affect the extremities, particularly the anterior legs, and the interphalangeal joints in a symmetrical pattern [7].

A comprehensive history and examination should be performed on patients with oral lichen planus in order to look into any possible extraoral lesions. The necessity for thorough assessment and interdisciplinary approaches to this illness is highlighted by the descriptive study involving 274 patients by Juliana et al. that had 37 oral lichen planus patients with concurrent involvement at several sites [8]. In our case, the patient had hypertrophic lichen planus as well as oral reticular lichen planus.

TND is more common in adults than in children; it is often referred to as trachyonychia, and it has no gender predilection. It is a term used to characterize a range of abnormalities in the nail plate surface that lead to rough nails. Loss of luster, bluish-brown staining, thinning of nail plates, longitudinal ridging, distal notching, and splitting are its characteristic features [9,10]. Trachyonychia is characterized by excessive longitudinal ridging. Even if only one nail is involved, it is still considered TND, and there is no need to affect all 20 nails. Nail dystrophy can appear all at once in all of the nails or it may appear gradually over the course of time [10]. TND differs from typical nail involvement in lichen planus because it is monomorphic, i.e., it affects the nail plate surface uniformly, involving all 20 nails, and does not exhibit longitudinal fissures or pterygium that is seen in cases of nail lichen planus. While other lichen planus symptoms are usually absent in TND, clinical nail changes in trachyonychia are comparable to those seen in nail lichen planus [10]. Our patient had nail plate thinning, longitudinal ridging, loss of luster, and subungual hyperkeratosis in toenails, which all are characteristic features of trachyonychia.

TND may result from a number of inflammatory conditions that disrupt the keratinization of the nail matrix, including lichen planus, psoriasis, eczema, pemphigus vulgaris, and alopecia areata. Alopecia areata is characterized by frequent fine nail pitting, which resembles the glossy nails observed in TND. Patients with psoriasis will exhibit uneven pitting and increased nail plate thickness in addition to the usual histological findings of psoriasis. Patients with eczema will have nail involvement in the form of transverse ridging, thickening, and discoloration of nails along with skin lesions on the palms and soles. There is a lack of clinical criteria to differentiate between inflammatory skin disorders and trachyonychia [6].

There are various options available for the treatment of lichen planus and trachynonychia; topical steroids are the first-line agents for cutaneous lichen planus. In addition to topical corticosteroids, a number of second-line treatments for lichen planus have been granted approval. Systemic glucocorticoids, phototherapy with ultraviolet B and ultraviolet A radiation, and oral retinoids are examples of second-line therapies. However, just like with topical corticosteroids, the available information in the literature suggests that these medications are either insufficient to treat lichen planus on their own or that their long-term use may pose safety risks. In particular, chronic systemic glucocorticoid use has been linked to major adverse effects like raised blood pressure and early atherosclerosis disease, as well as an increased risk of thinning of skin and ecchymoses. The better safety profile offered by JAK inhibitors holds promise for the prospective use of these medications in the management of lichen planus with greater response and early remission of disease, especially given the side effects of corticosteroids in the treatment of lichen planus. Since trachyonychia and mucocutaneous lichen planus coexisted in our case, we decided to treat the patient with tofacitinib, which can be given in all types of lichen planus [11].

## Conclusions

TND is considered a rare condition that usually presents in isolation without any systemic disease; however, in our case, it was associated with nail and mucocutaneous lichen planus. While trachyonychia presenting as one of the manifestations of nail lichen planus is often reported, mucocutaneous involvement with trachyonychia is an uncommon finding. It is thus important to perform a physical oral examination as well as a thorough cutaneous examination to look for other dermatoses associated with trachyonychia. Lichen planus, which is an acquired immune-mediated dermatosis, involves the skin, nails, oral mucosa, genital mucosa, and scalp. Hence, all the possible sites and areas should be examined properly and treated accordingly. Although it begins as an intensely pruritic disease, the patient's quality of life is often affected, leading to psychological distress, and hence early identification and treatment of lichen planus is important.

## References

[1] Weston G, Payette M (2015). Update on lichen planus and its clinical variants. Int J Womens Dermatol.

[2] Vičić M, Hlača N, Kaštelan M, Brajac I, Sotošek V, Prpić Massari L (2023). Comprehensive insight into lichen planus immunopathogenesis. Int J Mol Sci.

[3] Goettmann S, Zaraa I, Moulonguet I (2012). Nail lichen planus: epidemiological, clinical, pathological, therapeutic and prognosis study of 67 cases. J Eur Acad Dermatol Venereol.

[4] Jacobsen AA, Tosti A (2016). Trachyonychia and twenty-nail dystrophy: a comprehensive review and discussion of diagnostic accuracy. Skin Appendage Disord.

[5] Wechsuruk P, Bunyaratavej S, Kiratiwongwan R, Suphatsathienkul P, Wongdama S, Leeyaphan C (2021). Clinical features and treatment outcomes of nail lichen planus: a retrospective study. JAAD Case Rep.

[6] Gordon KA, Vega JM, Tosti A (2011). Trachyonychia: a comprehensive review. Indian J Dermatol Venereol Leprol.

[7] Cassol-Spanemberg J, Blanco-Carrión A, Rodríguez-de Rivera-Campillo ME, Estrugo-Devesa A, Jané-Salas E, López-López J (2019). Cutaneous, genital and oral lichen planus: a descriptive study of 274 patients. Med Oral Patol Oral Cir Bucal.

[8] Gorouhi F, Davari P, Fazel N (2014). Cutaneous and mucosal lichen planus: a comprehensive review of clinical subtypes, risk factors, diagnosis, and prognosis. ScientificWorldJournal.

[9] Nandedkar-Thomas MA, Scher RK (2005). An update on disorders of the nails. J Am Acad Dermatol.

[10] Makkar M, Pandey P, Dixit A, Kapur K, Mahajan NC (2011). Twenty nail dystrophy associated with lichen planus in a child: a case report. Iran J Dermatol.

[11] Abduelmula A, Bagit A, Mufti A, Yeung KC, Yeung J (2023). The use of Janus kinase inhibitors for lichen planus: an evidence-based review. J Cutan Med Surg.

